# Oral microbial dysbiosis linked to worsened periodontal condition in rheumatoid arthritis patients

**DOI:** 10.1038/s41598-019-44674-6

**Published:** 2019-06-10

**Authors:** Jôice Dias Corrêa, Gabriel R. Fernandes, Débora Cerqueira Calderaro, Santuza Maria Souza Mendonça, Janine Mayra Silva, Mayra Laino Albiero, Fernando Q. Cunha, E. Xiao, Gilda Aparecida Ferreira, Antônio Lúcio Teixeira, Chiranjit Mukherjee, Eugene J. Leys, Tarcília Aparecida Silva, Dana T. Graves

**Affiliations:** 1 Faculty of Dentistry, Federal University of Minas Gerais, Belo Horizonte, MG Brazil; 2 Oswaldo Cruz Fundation, René Rachou Research Center, Belo Horizonte, MG Brazil; 3 University Hospital, Federal University of Minas Gerais, Belo Horizonte, MG Brazil; 4 Faculty of Dentistry, University of Campinas, Piracicaba, SP Brazil; 5 Faculty of Medicine of Ribeirão Preto, University of São Paulo, Ribeirão Preto, SP Brazil; 6 Penn Dental School, University of Pennsylvania, Philadelphia, PA USA; 7 The Ohio State University, College of Dentistry, Columbus, OH USA

**Keywords:** Rheumatoid arthritis, Microbiology, Periodontics

## Abstract

Rheumatoid arthritis (RA) is an autoimmune disease characterized by joint inflammation. Individuals with RA have a higher risk of periodontitis and periodontitis has been linked to RA through the production of enzymes by periodontal pathogens that citrullinate proteins. This linkage is supported by findings that periodontitis is associated with increased RA severity and treatment of periodontitis can improve the symptoms of RA. The possible mechanism for this association is through dysbiosis of the oral microbiota triggered by RA-induced systemic inflammation. We examined the RA status of subjects by measuring the number of tender and swollen joints, anti-citrullinated protein antibody and rheumatoid factor. Periodontal disease status and salivary cytokine levels were measured, and dental plaque analyzed by 16S rRNA high throughput sequencing. RA patients had a higher bacterial load, a more diverse microbiota, an increase in bacterial species associated with periodontal disease, more clinical attachment loss, and increased production of inflammatory mediators including IL-17, IL-2, TNF, and IFN-γ. Furthermore, changes in the oral microbiota were linked to worse RA conditions. Our study provides new insights into the bi-directional relationship between periodontitis and RA and suggest that monitoring the periodontal health of RA patients is particularly important.

## Introduction

The oral cavity is the second largest microbial niche after the gastrointestinal tract with over 700 bacterial species^1^. In periodontally healthy individuals, microbial populations co-exist in equilibrium with the host. The change in this equilibrium is linked to the pathogenesis of oral diseases such as periodontitis^1,2^. Oral bacteria, which exist as a biofilm on the tooth surface, can induce inflammation in the adjacent gingiva, leading to osteoclast formation and bone loss which, in severe cases causes tooth loss^3^. Systemic inflammatory diseases may contribute to disrupting the balance between host and oral microbiota^4^.

Rheumatoid arthritis (RA) is a systemic autoimmune disease characterized by chronic inflammation and damage to articular tissues^5^. An increased incidence of periodontitis has been reported in patients with RA^6^. Furthermore, treatment of periodontitis has been shown to reduce RA activity^7^. Both conditions exhibit similar pathological features, including bone resorption^8–10^. It has been proposed that periodontal disease and RA are linked by periodontal pathogens that produce enzymes capable of modifying proteins to enhance their antigenicity through the addition of malondialdehyde-acetaldehyde, citrullination and carbamylation^8,10,11^. These anti-cyclic citrullinated peptide (CCP) antibodies can be detected before RA onset and have been identified as an etiologic factor in the disease process^12^. Furthermore, RA enhance systemic inflammation which can amplify the local inflammatory response in the periodontium, increasing periodontal destruction^13^.

Few studies have described the composition of the oral microbiota in patients with RA^14–17^. Zhang examined dental and salivary microbiome but the periodontal status of RA subjects was not defined^15^. Another study evaluated subgingival microbiota and the periodontal condition of RA subjects, but did not evaluate the impact of RA and periodontitis independently^14^. Mikuls compared RA to osteoarthritis patients^16^ while Lopez-Oliva analyzed only RA patients without periodontitis^17^. Furthermore, neither of these studies assessed inflammatory parameters in the oral cavity. Our study characterized the subgingival microbiome of RA patients and its association with periodontal status, inflammatory markers and RA scores to establish links between these parameters.

## Results

### Periodontal destruction and RA outcomes

Table 1 shows that of the 42 patients with RA included in the study, 21 (50%) had periodontitis compared to 20 (42.6%) of 47 non-RA subjects (P > 0.05). The mean duration of RA was similar for patients without periodontitis (16.18 ± 8.2 years) and for those with periodontitis (12.46 ± 9.7years) (P > 0.05). RA activity parameters (number of tender and swollen joints, DAS-28) and medications in use were not different between RA patients with or without periodontitis (Table 1). Interesting, the majority of RA subjects (85.7%) with periodontitis were positive for the presence of autoantibodies (ACPA) compared to only 33% in RA patients without periodontitis (P < 0.05).

**Table 1:** Demographic and clinical data of patients with RA and healthy control subjects.

	Controls		RA	
	Non-CP	CP	Non-CP	CP
Subjects	27 (57.4%)	20 (42.6%)	21 (50%)	21 (50%)
Females %	36.5	48	53.48	34
Age, years	42.8 (±14.0)	46.5 (±12.3)	50 (±11.1)	53 (±10.40)
Current Smokers	1 (3.7%)	2 (7.6%)	1 (4.6%)	1 (4.6%)
RA duration, years	n/a	n/a	16.2 (±8.2)	12.5 (±9.7)
**Disease active parameters**				
Tender joints	n/a	n/a	3.2 (±4.0)	3.3 (±4.6)
Swollen joints	n/a	n/a	2.5 (±0.5)	2.4 (±0.9)
DAS28	n/a	n/a	3.5 (±1.2)	3.7 (±1.5)
**Autoantibody status**				
ACPA positive, %	n/a	n/a	7 (33%)	85.7*
**Medications**				
Methotrexate	n/a	n/a	11 (52.4%)	14 (66.7%)
Prednisone	n/a	n/a	9 (42.9%)	14 (66.7%)
Biological agent	n/a	n/a	5 (23.8%)	4 (19.0%)
**Periodontal parameters**				
PD (mm)	1.9 (1.6–2.2)	3.0 (3–3.7)*	2.9 (2–3)^#^	3.8 (3.4–4.5)*^,#^
CAL (mm)	2.2 (2–3)	3.0 (2.6–3.5)	3.0 (2.9–3.4)	4.0 (3.7–5.3)*^,#^
BOP (% sites)	6 (1.2–16)	6.7 (2.7–13)	5 (3.8–7.7)	7 (5–14)
Missing teeth	2 (0–6)	4 (1–7)	6 (2.5–11)^#^	6 (3–10)
Plaque Index	0.5 (0.1–1)	0.5 (0.3–0.8)	0.5 (0.2–0.7)	0.58 (0.23–1.1)
Tooth brushing (times/day)	2.85 (±0.93)	2.62 (±0.85)	2.82 (±0.5)	2.69 (±0.6)

Values were expressed in mean ± SD or median (25% percentile-75% percentile).

CP: Chronic Periodontitis, BOP: bleeding upon probing, PD probing depth, CAL clinical attachment level, DAS28: Disease Activity Score, ACPA anti-citrullinated protein antibody.

*Statistically different comparing Non-CP × CP within the same group.

^#^Statistically different comparing RA × Healthy Control group with the same periodontal status.

One Way ANOVA or Kruskal-Wallis test, p < 0.05.

The presence of RA was associated with worse periodontal parameters compared with control subjects: probing depth (Controls 3.0 × RA 3.8 mm) and clinical attachment loss (Controls 3.0 × RA 4 mm) (Table 1). These data indicate severe periodontitis in RA patients. However, self-reported hygiene habits, plaque index and bleeding on probing did not differ among RA and control subjects (P > 0.05) (Table 1).

### RA affects subgingival microbial load, richness and diversity

**Figure 1:**
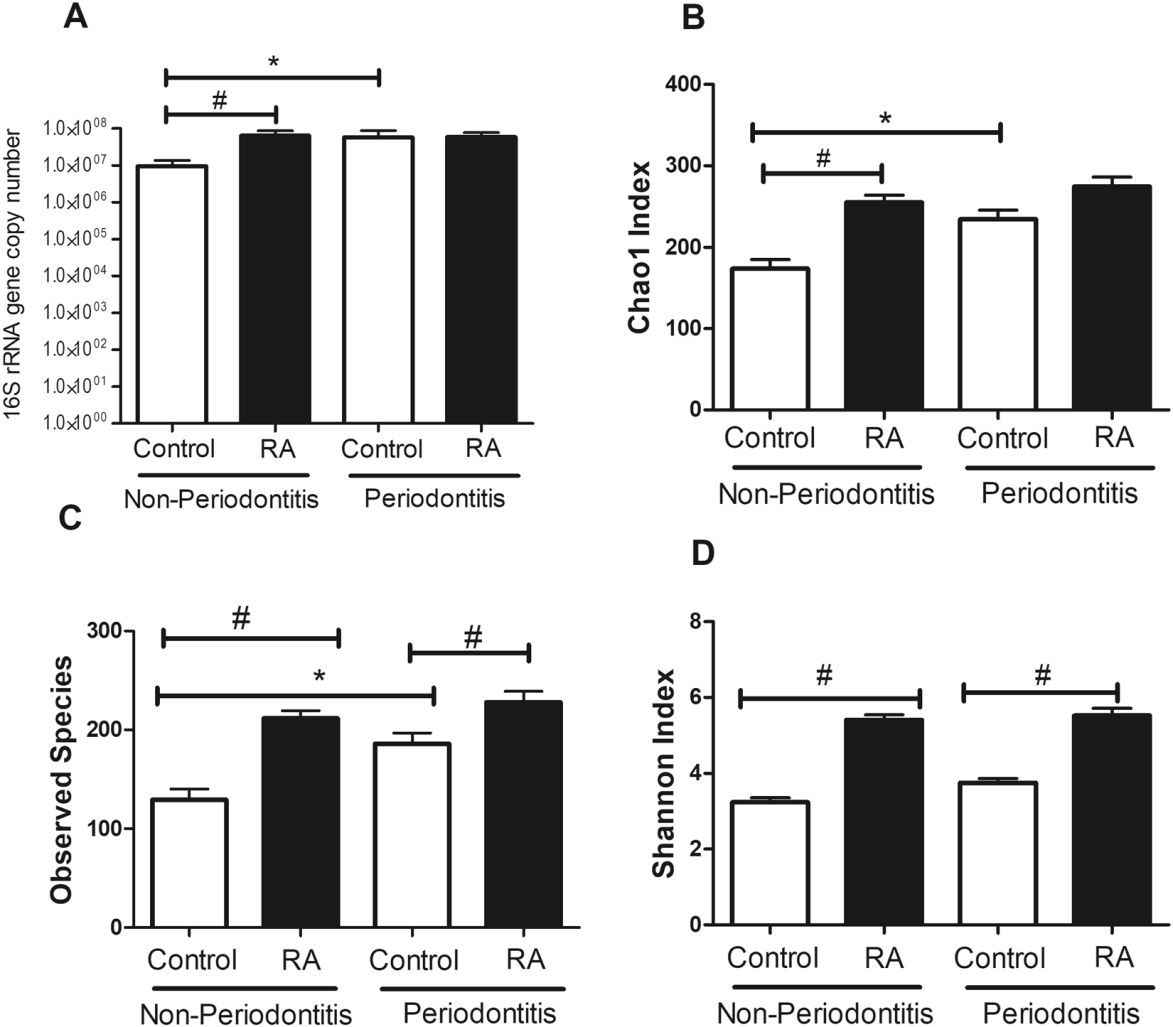
Bacterial load and microbial diversity in subgingival biofilm samples. (**A**) Bacterial load. (**B**–**D**) Metrics of alpha diversity in control subjects and RA patients with or without periodontitis. *Statistically different compared to non-periodontitis subjects within the same group. ^#^Statistically different compared to matched controls. p < 0.05, Kruskal-Wallis test.

We investigated the total microbial biomass in RA subjects and found that non-periodontitis RA patients had a significantly ~1 log higher bacterial burden than did control individuals without periodontitis (Fig. 1A). A total of 779 OTUS were found and the impact of RA status on microbial diversity and richness was examined by assessing the number of observed OTUs, Chao1 and Shannon indexes. RA patients had increased microbial diversity compared to controls. In subjects with periodontitis, RA was associated with increased diversity assessed by number of OTUs (Fig. 1C) and Shannon Index (Fig. 1D). In patients without periodontitis, RA caused a change in microbial diversity assessed by Chao1 index (Fig. 1B).

**Figure 2:**
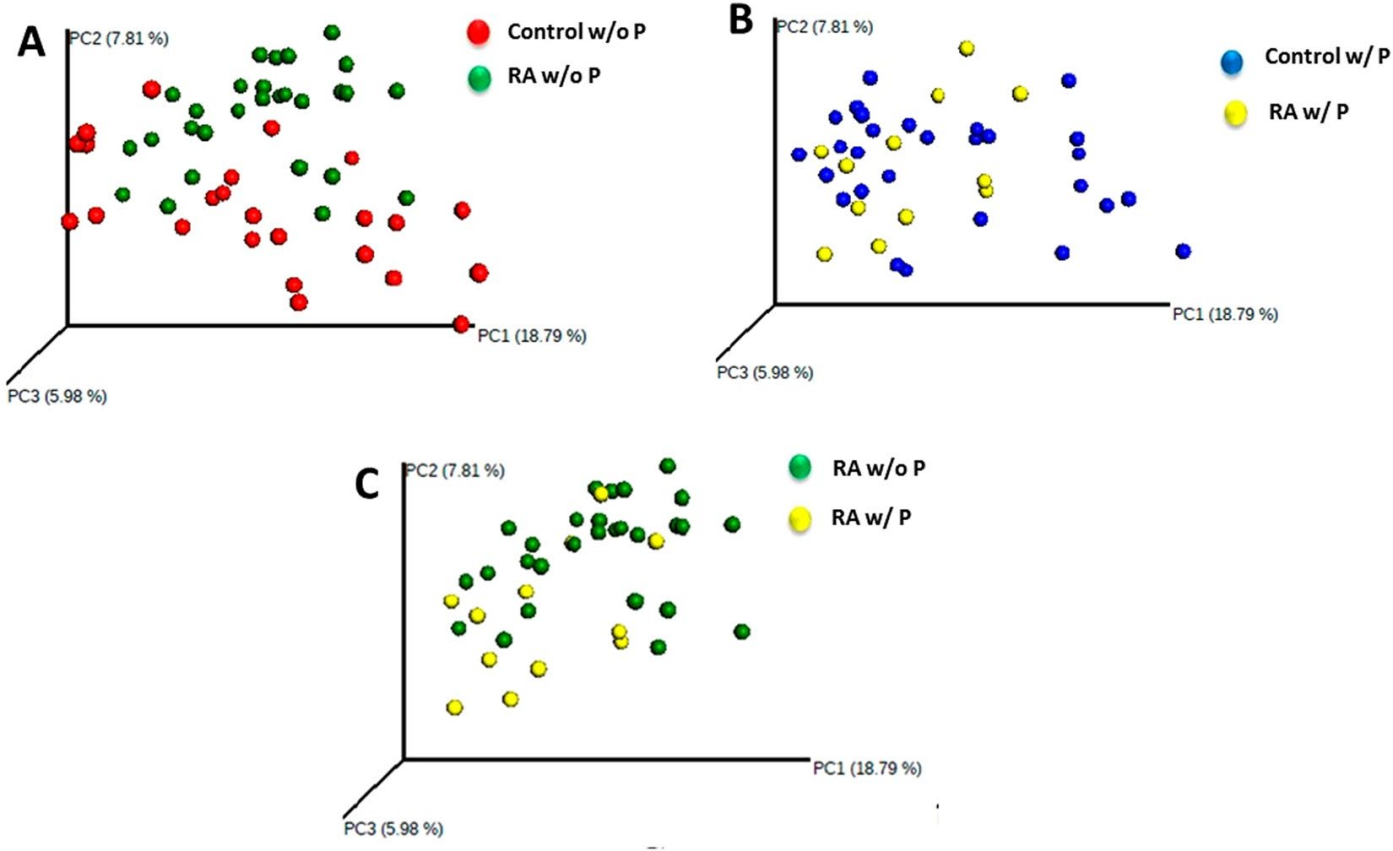
Principal coordinates analysis plots comparing subgingival microbial community compositions. Each point represents a subject. (**A**) Microbial communities in control and RA subjects without periodontitis. (w/o P). (**B**) Microbial communities in RA patients versus control subjects with periodontitis (w/P). (**C**) Microbial communities in RA patients without periodontitis versus with periodontitis.

To analyze whether the subgingival microbial communities in patients with RA were distinct from that of controls, we performed unweighted UniFrac distance analysis (Fig. 2). Microbial communities in patients with RA had distinct clusters compared to control patients without the complicating factor of periodontitis (Fig. 2A, PERMANOVA, p < 0.01). The presence of periodontitis in patients with RA obviated the difference between the RA and control group (P > 0.05, Fig. 2B). However, RA patients with periodontitis clustered separately from RA patients without periodontitis (P < 0.05, Fig. 2C).

### Subgingival RA and control group sites harbor distinct bacterial communities

**Figure 3:**
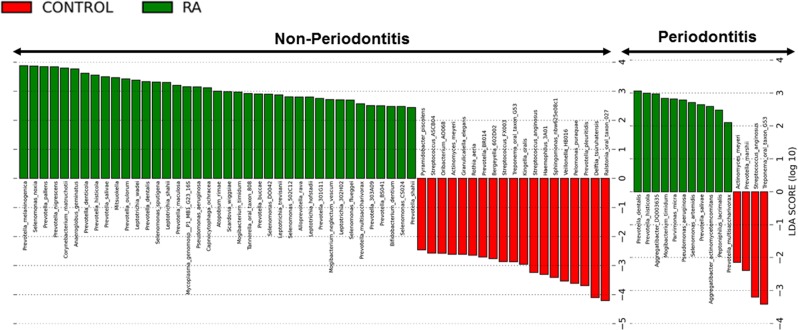
OTUs with different relative abundance based on LEfSe (linear discriminant analysis coupled with effect size measurements) results in control subjects (red) and RA subjects (green). (**A**) Subjects without periodontitis. (**B**) Subjects with periodontitis. Bars represent linear discriminant analysis scores (LDA).

We performed LEFSE (linear discriminant analysis coupled with effect size measurements) for analysis of the relative abundance of microbial taxonomic groups. A number of pathogenic bacteria were significantly elevated in the RA group that are associated with worse periodontal status^1^. RA patients without periodontitis had enrichment in periodontitis-associated bacteria such as *Prevotella* species (*P*. *melaninogenica*, *P*. *denticola*, *P*. *histicola*, *P*. *nigrescens*, *P*. *oulorum*, and *P*. *maculosa*) and other pathogenic species (*Selenomonas noxia*, *S*. *sputigena and Anaeroglobus geminatus*). In addition, RA subjects presented a reduction of health-associated species (*Streptococcus*, *Rothia aeria*, *Kingella oralis*, *Haemophilus*, *Actinomyces*) (Fig. 3A). In the same way, pathogenic species such as *Prevotella*, *Aggregaticbacter actinomycetemcomitans* and *Parvimonas micra* were significantly increased in RA patients with periodontitis compared to control subjects with periodontitis (Fig. 3B). We also observe an increased concentration of gram-negative anaerobic species on RA sites compared to control sites with periodontitis (Supplementary Fig. S1).

### RA parameters and oral bacteria species

Several bacterial taxa were correlated with RA parameters. In RA subjects with periodontitis, bacteria related to periodontal health, such as *Actinomyces*, were negatively correlated with number of tender joints (rho = −0.36, p < 0.05). On the other hand, in the same subjects, the presence of pathogenic species such as *Fretibacterium fastidiosum*^18^, *Parvimonas micra*^19^ and *Anaeroglobus geminatus*^1^ were correlated with augmented numbers of swollen (rho = 0.35) and tender joints (rho = 0.30), p < 0.05.

### Phylogenetic investigation of communities in subgingival microbiota in RA patients

To identify functional gene families in bacteria that may be differentially enriched in the subgingival microbiota of control subjects and RA patients, we applied PICRUSt (phylogenetic investigation of communities by reconstruction of unobserved states), a computational algorithm that provides a functional-gene profile for each sample based on a prediction of the number of copies of each gene family found in the 16S rRNA sequence information. Analysis using PICRUSt identified a number of genes involved with energy metabolism, lipopolysaccharide (LPS) biosynthesis, amino acid and carbohydrate metabolism, cell cycle and peptidases that were significantly more abundant in the subgingival bacteria of subjects with RA independent of periodontal status. In controls, bacterial genes involved with amino acid biosynthesis, and carbohydrate metabolism were over-represented in the periodontal microbiota (Supplementary Fig. S2).

### Salivary concentration of inflammatory cytokines in RA patients

**Figure 4:**
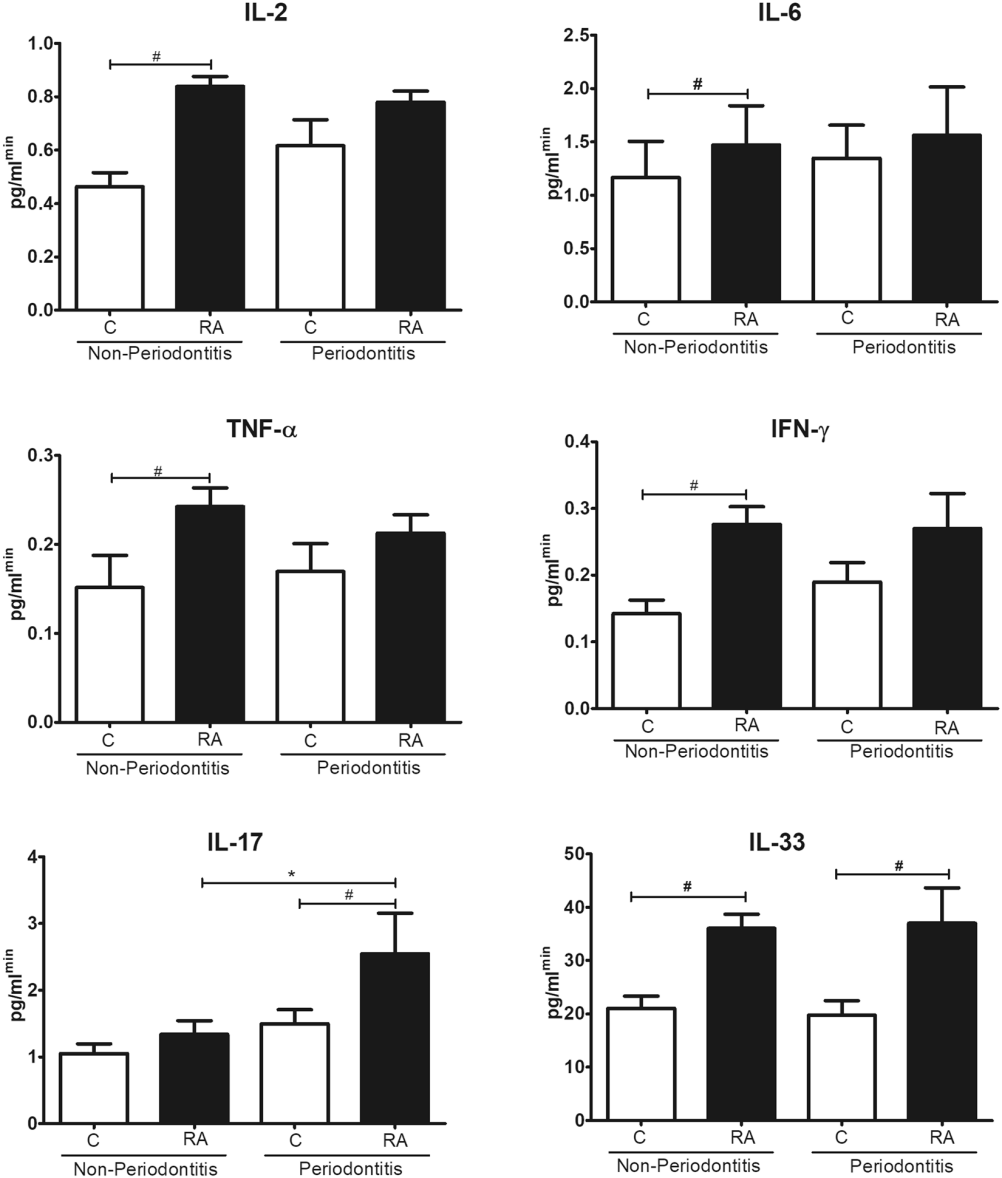
Levels of inflammatory cytokines in saliva. Control subjects and patients with rheumatoid arthritis (RA) with and without periodontitis. Cytokines were measured by cytometric bead array (CBA) or enzyme-linked immunosorbent assay (ELISA). *Statistically different compared to subjects without periodontitis within the same group. ^#^Statistically different compared to Control. p < 0.05, Kruskal-Wallis test.

To investigate whether the above-mentioned dysbiosis in subgingival microbiota could be associated with an altered inflammatory response we measured cytokines in saliva of RA and control subjects (Fig. 4). The levels of IL-2, IL-6 and IFN-γ were increased in saliva from RA patients compared to control subjects, both without periodontitis (P < 0.05). IL-33 and TNF-α were increased in all RA groups independent of periodontal status (P < 0.05). IL-17 was increased in RA subjects with periodontitis compared to control subjects (P < 0.05, Fig. 4).

The increased levels of the cytokines IL-6, IL-17 and IL-33 were positively correlated with periodontal parameters such as probing depth and number of missing teeth as demonstrated in Table 2 (P < 0.05). IL-33 levels were positively correlated with RA parameters such as rheumatoid factor and c-reactive protein (CRP), while IL-6 was positively correlated with CRP and ESR. Furthermore, the presence of healthy-related species including *Streptococcus*, *Rothia aeria*, *Actinomyces* was negatively correlated with cytokines IL-17 and TNF-α. In contrast, the presence of pathogenic species, such as *Selenomas* and *Prevotella*, were correlated with increased levels of inflammatory cytokines (IL-2, IL-6, IL-17, IL-33 and TNF-α) (Table 2).

**Table 2:** Correlations among inflammatory cytokines in saliva, relative abundance of bacteria, RA and Periodontal parameters in RA patients (rho values).

	IL-33	IL-2	TNF-α	IL-6	IL-17
**RA parameters**					
RF	0.80	—	—	—	—
CRP	0.60	—	—	0.32	—
ESR	—	—	—	0.50	—
**Periodontal parameters**					
PD (mm)	—	—	—	—	0.54
CAL (mm)	—	—	—	—	—
BOP (%)	0.51	—	—	—	—
Missing teeth	—	—	—	0.35	—
**Bacteria**					
*Rothia aeria*	—	—	—	—	−0.30
*Streptococcus*	—	—	−0.46	—	−0.35
*Actinomyces*	—	—	−0.45	—	−0.41
*Haemophilus*	−0.35	—	—	—	—
*Selenomonas*	0.32	—	—	0.38	—
*Selenomonas noxia*	0.35	—	—	0.35	—
*Prevotella oralis*	—	0.30	0.31	0.47	0.30
*Fusobacterium nucleatum*	—	—	—	0.50	0.35

BOP: bleeding upon probing, PD: probing depth, CAL: clinical attachment level, CRP: C-reactive protein; IL: interleukin; ESR: Erythrocyte sedimentation rate, RF: rheumatoid factor; IL: interleukin; TNF: tumor necrosis factor. All values showed were statistically significant at value of p < 0.05, Spearman rank correlation.

### Subgingival biofilm of RA patients elicited high IFN-γ production from PBMCs

To evaluate the potential of dysbiotic subgingival biofilm of RA patients to stimulate inflammatory response, we exposed PBMCs from control individuals to inactivated bacterial plaque samples from RA or healthy donors, neither of which had periodontitis. Plaque samples from RA subjects’ stimulated significantly higher production of IFN-γ compared to plaque from control subjects (Supplementary Fig. S3).

## Discussion

A relationship between RA and periodontitis has previously been reported, but the impact of RA on the subgingival microbiota linked to periodontal disease has not been thoroughly investigated and mechanisms for the potential impact have not been addressed^20–25^. In the present study, we showed significant differences in the subgingival bacterial community between RA patients and controls. RA patients had a higher bacterial load, a more diverse microbiota and increased abundance of pathogenic species compared to controls, even in periodontally healthy individuals. Accordingly, periodontal destruction (probing depth and clinical attachment loss) was significantly greater in RA subjects.

Microbiota homeostasis can be modulated by a number of parameters including genetic, environmental and inflammatory factors^26^. Chronic systemic inflammation, as observed in RA, may affect the levels of inflammation in periodontal tissues, which in turn may alter the bacterial community. IL-2, IFN-γ, TNF and IL-33 exhibited an approximately two-fold increase in RA subjects without periodontitis compared to matched controls. IL-6 had a significant although less robust change. Taken together these results indicate that RA impacts the cytokine levels in periodontal tissues reflecting increased inflammation. Similar changes in periodontal ifnlamation have been reported in subjects with systemic lupus erythematosus^27^ and RA^28^. Several studies report a positive correlation between salivary cytokine levels and periodontitis^29,30^ while some have not^31^. The discrepancy may be due to different stages of disease, different disease classification systems, methodological differences and that whole saliva does not simply represent health versus diseased sites but reflects the aggregate of healthy, gingivitis and periodontitis sites making the analysis more complex. IFN-γ is a Th1 cytokine that enhance cell-mediate responses^32^. TNF, IL-6 and IL-17 have multiple overlapping functions that contribute to RA and periodontal disease by stimulating leukocyte activation and migration, chemokine expression and bone resorption^23,33^. IL-33 stimulates Th2 responses^34^, while IFN-γ and IL-2 regulate several aspects of the immune response^35^.

The increased inflammatory cytokine levels in RA subjects was reflected by increased inflammatory potential of the bacteria from these individuals as reflected by greater stimulation of IFN-γ in PBMCs from inactivated dental plaque compared to stimulation by dental plaque from matched control subjects. Previous studies have suggested that periodontitis and RA are interdependent with elevated levels of pro-inflammatory molecules in both^36^. Inflammatory mediators found in the subgingival microenvironment may change the ecological conditions to favor the outgrowth of pathogenic bacteria, leading to periodontal destruction^37^ as shown for diabetes^38^. The increased inflammation caused by RA coupled with microbial changes may amplify periodontal inflammation and explain the greater su sceptibility to periodontitis in RA subjects that we and others have observed^39,40^. Studies in mice showed that RA is linked to greater periodontal bone loss and changes in the oral microbiota^24^. Our results provide evidence that similar events occur in humans.

We found that RA was associated with increased microbial load and diversity, which is consistent with previous reports that periodontitis, unlike most polymicrobial infections, is associated with increased bacterial diversity^41^. In addition, we observed that the severity of RA, reflected by the number of tender and swollen joints, was significantly correlated with the presence of pathogenic oral bacteria (i.e. *Fusobacterium nucleatum* and *Treponema socransky*). RA patients without periodontal disease have previously been reported to have increased bacterial biomass in their dental plaque, consistent with our results^17^ and bacterial changes in individuals with RA have been linked to parameters of RA severity. We examined samples that reflected similar sites within the same individual and our analysis focused on differences between individuals. However, this does not rule out the possibility that within an individual there are differences based on the periodontal status of a specific tooth.

Increased numbers of gram-negative anaerobic species are associated with proteolysis, inflammation and periodontal destruction^42^. Our analysis of predicted functions using PICRUSt identified an enrichment of several pathways linked to amino acid metabolism and peptidases in the microbiota from RA subjects. We also observed an abundance of genes linked to bacterial virulence such as LPS and sporulation, with the caveat that PICRUST analysis is descriptive, predicting genes that are present in the biofilm, but not necessarily genes that are expressed. These peptidases and virulence factors may increase inflammation and contribute to periodontal breakdown^43^.

Local changes in the periodontal tissues associated with enhanced inflammation can alter the environment and promote the growth of pathogenic bacteria, amplifying inflammation^40,44^. Subgingival sites in RA patients had an increase in gram-negative anaerobic species in dental plaque that are strongly associated with periodontal destruction and inflammation. We found higher levels of *Selenomonas noxia* and *Parvimonas micra* in RA subjects in agreement with previous findings in animal model of RA^24^ as well as elevated levels of *Prevotella*. A recent study found an association between anti-citrullinated peptide antibody (ACPA) titers and evidence of greater exposure to *Prevotella*^45^. ACPAs may be regarded as etiologic factors and diagnostic markers as they are detected in the serum before the disease onset. It is also possible that ACPAs represent different forms of RA^46^ and that ACPA-positive patients have a worse clinical prognosis with higher rates of erosive damage^47^. Here we observed that most of the RA patients with periodontitis (85%) were ACPA-positive. In agreement with this finding, ACPA-positive patients have a higher incidence of periodontitis than patients with osteoarthritis^48^ and RA patients with periodontitis exhibit high titers of ACPAs and greater periodontal inflammation^49,50^.

Recently our group reported the impact of another systemic autoimmune disease, systemic lupus erythematosus (SLE) on periodontal status and subgingival microbiota^51^. Patients with SLE exhibit a higher prevalence of periodontitis, which occurs at a younger age when compared to healthy individuals. Like RA, SLE caused an increased bacterial load in subgingival sites and induced changes in the microbial composition and diversity that were linked to increased cytokines concentration on saliva (IL-6, IL-17 and IL-33), similar to the changes observed for RA patients. Together these results corroborate the hypothesis that systemic inflammatory conditions are associated with dysbiosis of subgingival microbiota and increased risk of periodontitis.

A limitation of our study is its cross-sectional design with a single time measurement. Therefore, we are unable to answer the question whether changes in the microbiota are a cause and/or effect of the RA. In addition, the use of different medications to treat RA may affect periodontal inflammation and the microbiota. Despite these limitations that data presented is significant. Our study is the first to demonstrate the influence of RA on subgingival microbiota taking into account the periodontal status of the subjects.  The results support the concept that RA is an inflammatory disease that can modulate the subgingival biofilm and thus increase susceptibility to periodontal diseases.

## Methods

### Subjects

During one year we evaluated patients from the Rheumatology Outpatient Clinic of Clinics Hospital of Federal University of Minas Gerais (UFMG), Belo Horizonte, Brazil that were diagnosed with RA based on the 2010 American College of Rheumatology and EULAR classification criteria^52^. Two hundred thirty-nine patients agreed to participate, however, the study group consisted of forty-two patients based on the following inclusion criteria: no other rheumatic disease, no treatment for periodontal disease within the last 6 months, no use of orthodontic appliances, no use of antibiotics within the last 3 months, no pregnancy or lactation and the presence of at least 8 teeth. The control group consisted of 47 subjects without RA or other rheumatic diseases, that were age and gender matched with the RA group, randomly assigned from a population with demographic, social, and educational backgrounds similar to RA patients. Their medical history was obtained from an interview. Patients’ medical history and medications were determined by review of medical charts. Each patient had laboratory assessments that were taken from the medical record which included systemic measures of RA including blood levels of IgM rheumatoid factor (RF) and anti–citrullinated protein antibody (ACPA). As a measure of systemic inflammation C-reactive protein (CRP) was assessed as well as erythrocyte sedimentation rate (ESR). Periodontal status was assessed by two calibrated examiners (JDC and SMM) and the following parameters were recorded: plaque index, probing depth, clinical attachment level and bleeding on probing. Periodontitis was defined as the presence of two or more interproximal sites with probing depth ≥4 mm or one site with probing depth ≥5 mm^53^.

### Ethics statement

The subjects gave written informed consent, and the study protocol was approved by the Federal University of Minas Gerais Ethics Committee (CAAE: 03128012.0.0000.5149/2012). All patient data and subgingival samples were anonymized. For sampling of PBMCs from the blood of healthy subjects they also gave written informed consent and their data were anonymized. All methods used here were carried out in accordance with the relevant guidelines and regulations.

### Subgingival samples collection

Subgingival samples were collected using endodontic paper points (ISO40) (Tanariman, Manacaparu, AM, Brazil) that were inserted in 5 sites with deepest probing depth for one minute. After removal, the material was pooled together and stored in a sterile tube containing 500 µL of sterile distilled water and centrifuged at 3,000 g for 5 minutes. The paper points were discharged, and the pellet was kept at −80 °C until DNA extraction.

### Saliva collection

Saliva was collected by continuous drooling into a sterile 50 mL tube for 5 minutes. The salivary flow was measured in milliliters per minute (ml/min). The saliva samples were diluted (1:1) in a phosphate-buffered saline (PBS) solution containing protease inhibitors and subsequently frozen at −80 °C until analysis.

### Cytokines measurement

Concentrations of interleukin-2 (IL-2), IL-6, IL-17, tumor necrosis factor-α (TNF-α and interferon-γ (IFN-γ) in saliva were determined using a Cytokine Bead Array (CBA) Human Th1/Th2/Th17 Kit (BD Biosciences, San Diego, CA) and analyzed on a BD FACS Calibur flow cytometer (BD Biosciences). The concentration of IL-33 was measured by enzyme-linked immunosorbent assay (ELISA) (R&D Systems, Minneapolis, MN, USA). Assays were performed according to the manufacturer’s instructions. The results were expressed as picograms of cytokine (pg/ml) adjusted according to salivary flow.

### DNA extraction and sequencing

DNA was extracted from plaque samples using the Quick-g DNA MicroPrep kit (Zymo Research, Irvine, CA, USA) and 50 µL (10 mg/ml) of lysozyme per sample to enhance bacterial lysis as described^54^. The quantity and quality of DNA was measured spectrophotometrically (Tecan, Männedorf, Switzerland). The primers 515F (5′-GTGCCAGCMGCCGCGGTAA-3′) and 806R (5′-GGACTACHVGGGTWTCTAAT-3′) which target the hypervariable V4 region of the 16S rRNA gene were used for amplification^55^. After, agarose gel electrophoresis was performed to check size integrity. All amplicons were subjected to Illumina MiSeq Platform at the Next-Generation Sequencing Core of University of Pennsylvania and sequenced together at the same run. All Illumina sequence data were submitted to the NCBI Sequence Read Archive (SRA) under BioProject accession number PRJNA325500.

### Microbiota analysis

The raw reads were trimmed to remove regions with a low Phred score. Trimmomatic^56^ was the tool used with the TRAILING:5 and SLIDINGWINDOW:4:15 parameters. The trimmed reads were merged using FLASH^57^ tool requiring 30 reads overlap. The assembled amplicons were mapped to the CORE database using the Qiime’s pick_closed_reference_otus and 97% identity threshold. The representative set of sequences had their taxonomic classification using the same database and the Qiime’s assign_taxonomy script. The alpha diversity indexes were assessed using the Vegan R package^58^. Beta diversity was calculated using Unique Fraction metric (UNIFRAC) unweighted. Quantification of total bacterial load was determined by real-time PCR using universal primers for 16S rRNA gene. (F: AGAGTTTGATCCTGGCTCAG; R: ACGGCTACCTTGTTACGACTT) (IDT, Coralville, Iowa, USA) based on a a standard curve prepared using DNA extracted from a known number of *Porphyromonas gingivalis* (colony forming units) separated with flow cytometry and amplified with the same qPCR protocol. Samples were assayed in duplicate in a 25 μl reaction mixture containing 2.5 μl of template DNA, 2.5 μl of 10x TaqMan Universal PCR Master Mix, 1.5 μl of MgCl_2_,1 μl dNTP, 12.5 pmol of forward primer and reverse primer. The standard curve was used to derive the Cq (quantification cycle value) vs log CFUs linear equation. The cycling conditions used were as follows: 95 °C for 10 min, followed by 40 cycles at 95 °C for 15 s and 60 °C for 1 min each.

To provide an inference of the functional profile of the microbial community based on 16 S rRNA gene sequence results we assigned taxonomy to the representative set of sequences using the GreenGenes 13.5 database. R. We utilized PICRUSt (phylogenetic investigation of communities by reconstruction of unobserved states)^59^, a computational approach to predict the functional composition of a microbial community using marker 16S sequencing data and a database of reference genomes, to identify and quantify the Pathways and KEGG Orthology Groups.

### Blood collection and *in vitro* induction of PBMCs with oral biofilm

Blood was obtained from 5 systemically healthy individuals that did not have periodontitis or current use of immunosuppressive or anti-inflammatory drugs. Peripheral blood mononuclear cells (PBMCs) isolated by Ficoll–Paque gradient (Amersham Biosciences, Uppsala, Sweden) for 40 minutes at 20 °C. PBMCs were washed twice with PBS and counted in a hemocytometer chamber. The PBMCs of each patient (10^6^ cells/ml) were incubated in a complete RPMI medium of 2 mM L-glutamine, 5% normal human serum, 100 l g/mL streptomycin, and 100 UI/mL penicillin G potassium with subgingival plaque in 96-well plates. For these assays total subgingival plaque was removed from a given site, placed in 100 ul of TE buffer, homogenized by vortexing and heat/freeze inactivated as described by Moutsopoulos and colleagues^60^. PBMCs were exposed to a standardized amount of inactivated bacteria (1 × 10^8^ CFU) for 24 h and then supernatants were processed to evaluate cytokine concentration.

### Statistics

After evaluating the normality of distribution by Kolmogorov-Smirnov tests, clinical, demographic, alpha diversity, cytokine levels and bacterial load data were compared using One Way ANOVA or Kruskal-Wallis test. Correlations between relative abundance of taxa and clinical parameters of periodontal disease and RA were calculated using Spearman correlation coefficients (SPSS software, version 20). PERMANOVA was performed to compare beta diversity (QIIME software, version 1.9). In case of multiple comparisons, the p-value was corrected using Bonferroni correction. The predicted functional groups, and Operational Taxonomic Units (OTU), were compared among RA and control subjects and tested for statistical significance using DESeq2^61^ (R statistical software, version 3.5) and LEFSE^62^, respectively. P values < 0.05 were statistically significant.

## Supplementary information

Supplementary figures

## Data Availability

All Illumina sequence data from this study were submitted to the NCBI Sequence Read Archive (SRA) under BioProject Accession Number PRJNA325500.
