# Supplementary material for: Oral microbial dysbiosis linked to worsened periodontal condition in rheumatoid arthritis patients
Source: Sci Rep. 2019 Jun 10;9:8379. doi: 10.1038/s41598-019-44674-6 (PMC6557833; doi:10.1038/s41598-019-44674-6)
Supplement: Supplementary file 1 — Supplementary figures [file 41598_2019_44674_MOESM1_ESM.pdf]

Oral microbial dysbiosis linked to worsened periodontal condition in rheumatoid arthritis patients

Jôice Dias CORRÊA[1], Gabriel R. FERNANDES[2], Débora Cerqueira CALDERARO[3], Santuza Maria Souza MENDONÇA[1], Janine Mayra Silva[1], Mayra Laino ALBIERO[4], Fernando Q CUNHA[5], E XIAO[6], Gilda Aparecida FERREIRA[3], Antônio Lúcio TEIXEIRA[3], Chiranjit MUKHERJEE[7], Eugene J. LEYS[7], Tarcília Aparecida SILVA[1¶], Dana T. GRAVES[6¶*]

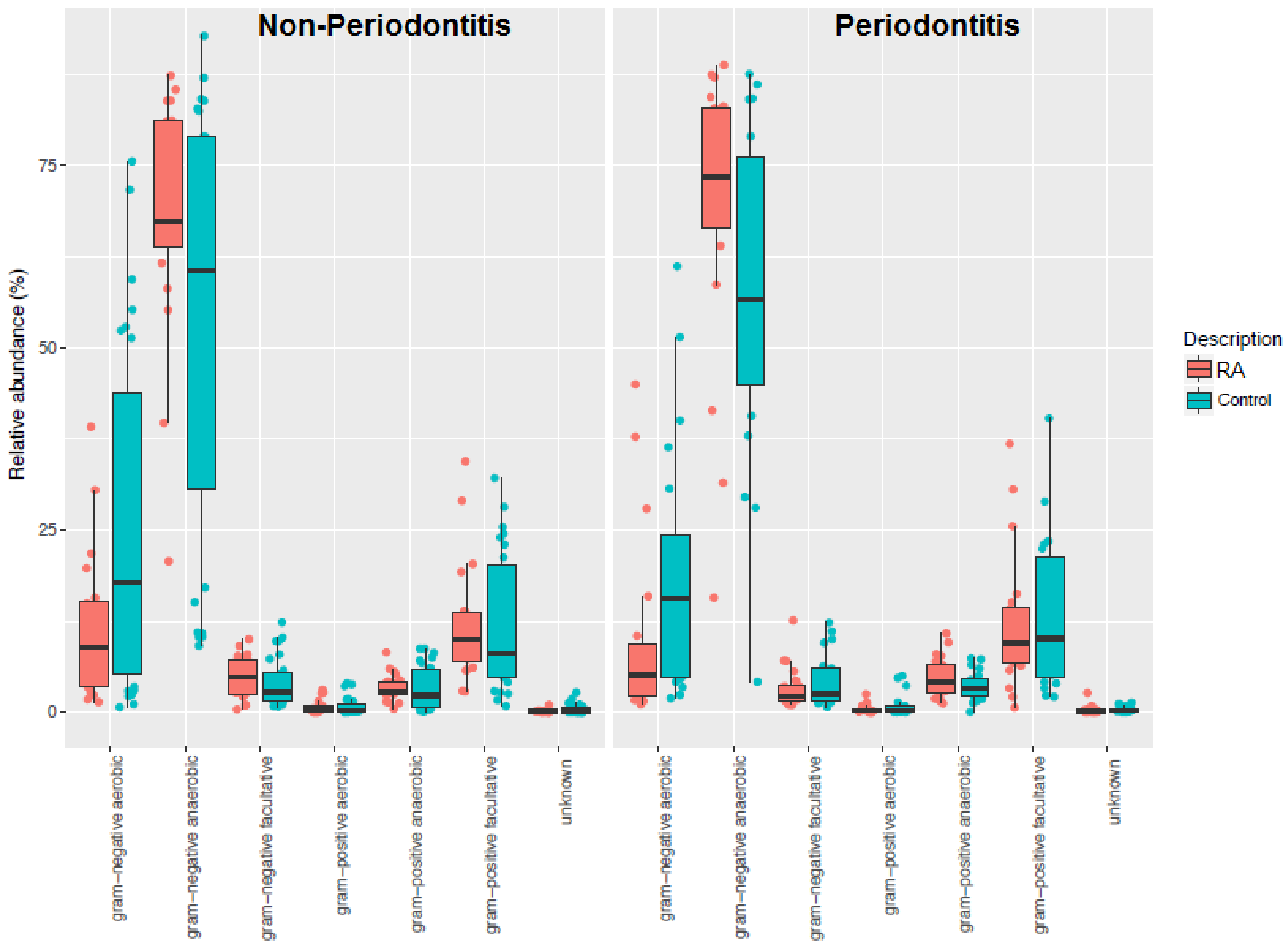

S1 Fig. – Relative abundance (%) of microbiota composition. Gram status and oxygen metabolism of 3 subgingival microbiota in RA patients and Control subjects, without and with periodontitis.

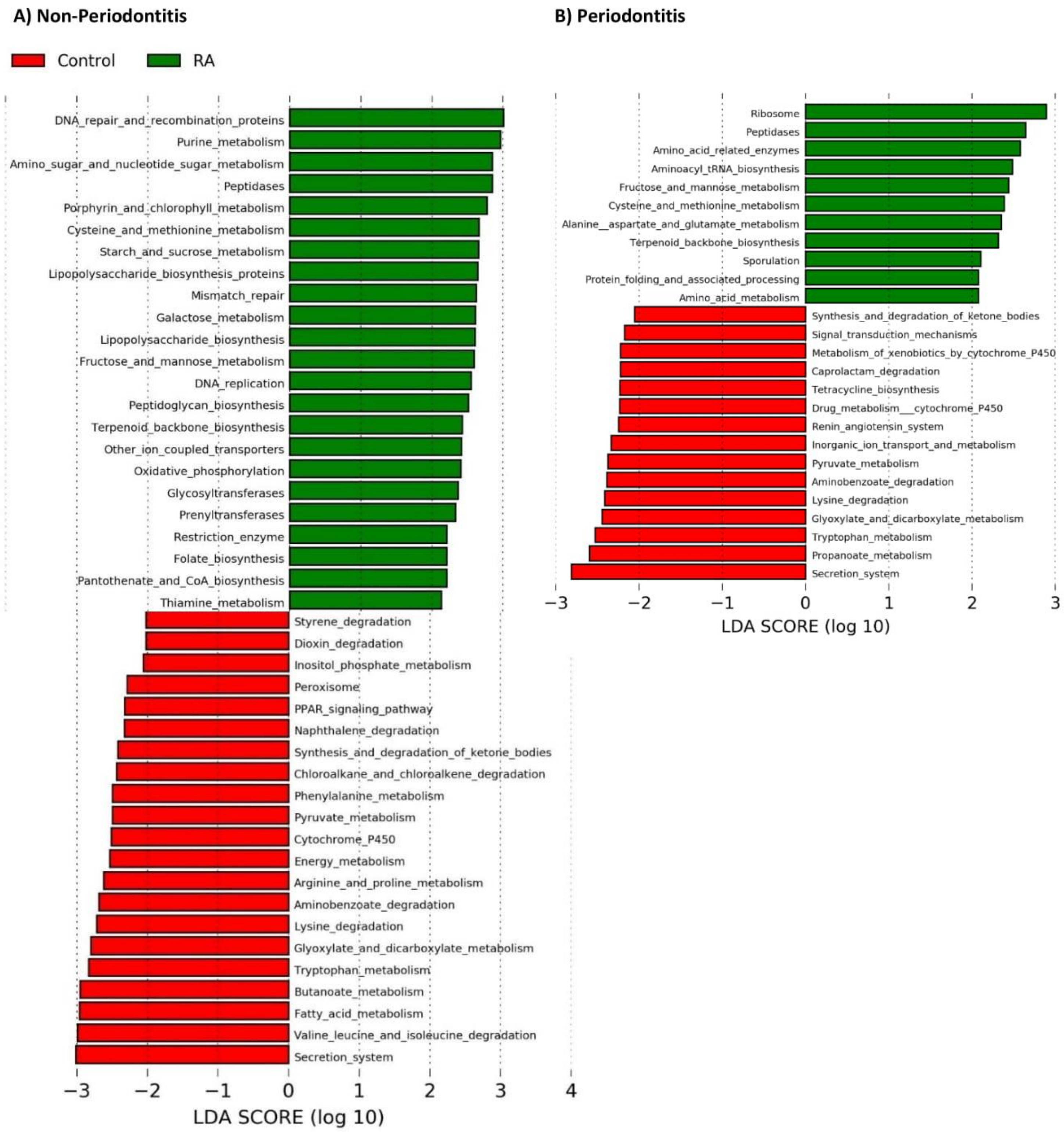

S2 Fig - Differentially abundant gene functions in subgingival microbiota. Control and RA subjects 6 without (A) and with periodontitis (B). Functional categories of genes of the subgingival metagenome were 7 predicted by using PICRUSt, and differentially abundant functions were then identified by using linear 8 discriminant analysis (LDA) coupled with effect size measurements (LEfSe).

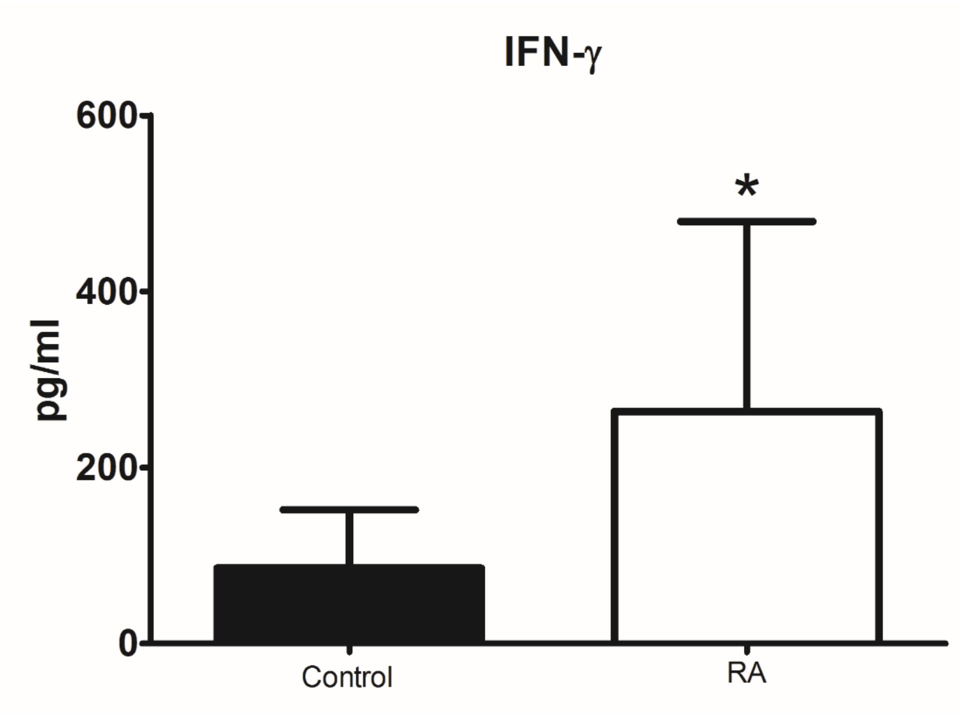

S3 Fig - Immunostimulatory potential of dental plaque from RA patients. Exposure of human PBMCs to 12 RA microbial plaque. Levels of IFN-□ was determined by ELISA. *statistically different compared to 13 Control. p<0.05, Student t-test.
